# Evaluation of Computer-Aided Navigation and Augmented Reality for Bicortical Mini-Implant Placement in Maxillary Expansion: An In Vitro Study

**DOI:** 10.3390/bioengineering12070703

**Published:** 2025-06-27

**Authors:** Giovanni Giovannini Riso, Javier Flores-Fraile, Gianmarco Perrone, Georgia Tzironi, Ana Belén Lobo Galindo, Cosimo Galletti, Álvaro Zubizarreta-Macho

**Affiliations:** 1 Faculty of Dentistry, Alfonso X el Sabio University, 28691 Madrid, Spain; giovanni@uax.es (G.G.R.); gperrone@uax.es (G.P.); amacho@uax.es (Á.Z.-M.); 2 Department of Surgery, Faculty of Medicine and Dentistry, University of Salamanca, 37008 Salamanca, Spain; georgiatzironi@usal.es (G.T.); alobogal@usal.es (A.B.L.G.); 3 Faculty of Medicine and Surgery, Kore University of Enna, 94100 Enna, Italy; cosimo.galletti@unikore.it

**Keywords:** orthodontics, mini-implants, palatal expansion, maxillary expansion, maxillary skeletal expansion, computer-aided navigation, augmented reality

## Abstract

The objective of the present study was to evaluate and compare the accuracy of the computer-aided static navigation technique (NAV), augmented reality (AR) and freehand placement technique (FHT) for the bicortical orthodontic self-drilling mini-implants for maxillary skeletal expansion (MSE) appliances placed in palate. **Material and Methods**: A total of 120 bicortical orthodontic self-drilling mini-implants were placed in the palate of ten 3D printed anatomically based polyurethane models of a completely edentulous upper maxilla. The orthodontic mini-implants were randomly assigned to the following placement techniques: (A) computer-aided static navigation technique (*n* = 40) (NAV), (B) augmented reality device (*n* = 40) (AR) and (C) conventional freehand technique (*n* = 40) (FHT). Moreover, two implants were placed in each side of the midpalatal suture in every model according to the digital planification of the expander device. Subsequently, the orthodontic mini-implants were placed and postoperative CBCT scans were performed. Finally, coronal entry-point (mm), apical end-point (mm) and angular deviations (°) were calculated using a *t*-test. **Results**: Statistically significant differences were shown at coronal entry-point (*p* < 0.001), apical end-point (*p* < 0.001) and angular deviations (*p* < 0.001) between the three placement techniques of bicortical orthodontic mini-implants. Additionally, statistically significant differences were also shown between the orthodontic mini-implant positions concerning the entry point (*p* = 0.004) and angular deviation (*p* = 0.004). **Conclusions**: The augmented reality placement technique results are more accurate, followed by the computer-aided static navigation technique and the freehand technique for MSE appliances placed in palate.

## 1. Introduction

Maxillary transverse deficiency is a common problem in orthodontics and may have several clinical manifestations, such as posterior crossbite, dental crowding, protrusion of incisors, accentuated curve of Wilson, and dark spaces in the buccal corridors [1]. These undesirable drawbacks have been commonly treated by rapid palatal expansion (RPE) procedures in growing patients [1,2,3]. RPE has been proved to be a simple and predictable therapeutic approach with stable long- and short-term results, regardless of the type of maxillary expander used during primary, mixed and early permanent dentition [4]. However mature patients are often subjected to more invasive techniques, such as surgically assisted rapid maxillary expansion (SARPE), due to palatine suture fusion. Recently it has been demonstrated that maxillary expansion is possible in grown patients using mini-implants placed in each side of the palatal suture instead of performing osteotomies. The above-mentioned procedure is called mini-implants-assisted rapid palatal expansion (MARPE) [5]. MARPE consists of a less invasive procedure, avoiding surgery and permitting a more parallel expansion, which is encouraging considering that SARPE leads to a V-shaped palatal opening [6].

Over the last decades, various types of MARPE techniques have been used with the form of different appliances, according to the preference of the clinician and the commercial availability [7]. Moon introduced an appliance that resembles the Hyrax expander, fitting closely into the palatal vault and incorporates four mini-implants which are used to fixate it to the palatal bone. As the appliance is activated, the force generated is more efficiently centered on the midpalatal suture [8]. Results have shown that this modification of Hyrax is both clinically accepted by the patient and provides stable results for young adults [9], although the retention period should be determined separately in every patient [10]. Despite MARPE’s increasing popularity, mini-implants placement comes with a risk. A variety of surgical guides has been introduced in order to allow precise insertion of mini-implants [11,12]. Cone beam computed tomography (CBCT) has been used successfully over the past few years to evaluate the sites of mini-implants placement in procedures of rapid maxillary expansion [13]. Furthermore, the use of advanced technology such as CBCT has increased due to the reduced cost and radiation dose for the patient compared to magnetic resonance imaging [14,15]. In addition, CBCT has been proven to be much more accurate in assessing mini-implant position, compared to panoramic and periapical radiographs which were commonly used in the past [16]. Therefore, CBCT consists of a useful tool to provide not only accurate measurement of palatal bone thickness, but also important information for the bone site most suitable for mini-implant placement [17].

In the past decades, the concept of guided implant surgery has been popularized due to its efficacy and precision allowing a more accurate and safer implant placement [18]. Before the era of digital technology, a diagnostic wax up was used on duplicated dental casts and over this wax up that was usually performed by dental technicians, a radiographic template was fabricated. Nowadays there are two types of digital techniques available: the ‘static’ technique, where digital templates are used to guide the surgeon in implant positioning, and the ‘dynamic’ technique, where the selected implant position is transferred to the surgical area via a navigation system [19]. Each of these methods has its pros and cons; for example, the static method is less flexible when it comes to changing of treatment plan, but it does not require expensive type of equipment, complicated software and has no space or time limitation. On the other hand, the dynamic technique can provide real time visual guidance during surgery [20].

Augmented reality is a term used to describe the technology that superimposes a computer-generated image on a user’s view in the real world, providing with a composing view. The visual objects are usually being obtained with three-dimensional X-rays and then are manipulated digitally with commercial software. Finally, the object is exported providing the user with visualization of digital data directly to the patient. Augmented reality has been used widely in the fields of dentistry and maxillofacial surgery and the results have been satisfactory for precision and avoidance of complications but not for reduction of operation time [21]. In orthodontics, augmented reality has been used for guided bracket positioning, as well as dental education [22,23].

The objective of the present study was to evaluate and compare the accuracy of the computer-aided static navigation technique, augmented reality and freehand placement technique for the bicortical orthodontic self-drilling mini-implants for MSE appliances, with a null hypothesis (H_0_) that there are no differences in the accuracy of the computer-aided static navigation technique, augmented reality and freehand placement technique for the bicortical orthodontic self-drilling mini-implants for MSE appliances.

## 2. Methods

### 2.1. Study Design

Researchers conducted a controlled experimental trial between January to March 2022 at the Dental Centre of Innovation and Advanced Specialties at Alfonso X El Sabio University in Madrid, Spain. The Ethical Committee of the Faculty of Health Sciences at Alfonso X El Sabio University approved the study in December 2021 (process No. 2/2022). In addition, this study was conducted in accordance with the ethical guidelines established by the Declaration of Helsinki and the CONSORT Statement. The 67-year-old patient provided informed consent for her preoperative CBCT scan to be used in this study. A power of 80.00% was calculated using the bilateral Student’s *t*-test for two independent samples. When used to calculate the variation from the null hypothesis H_0_: μ_1_ = μ_2_, the significance level of 5.00% and power of 80.00% meant that one hundred and twenty bicortical orthodontic self-drilling mini-implants were necessary for the purposes of this study.

### 2.2. Experimental Procedure

One hundred and twenty (120) bicortical orthodontic self-drilling mini-implants (Biomaterials Korea, Seoul, Republic of Korea) with 1.8 mm diameter [24,25,26] and 9 mm length were planned [27] and placed in anatomically based standardized polyurethane models of a completely edentulous upper maxilla, manufactured using a 3D impression procedure (Sawbones Europe AB, Malmo, Sweden) and based on a preoperative CBCT scan (WhiteFox, Satelec, Merignac, France). The CBCT scan was taken from a real patient using the following exposure parameters: 8.0 mA, 105.0 kV peak, 7.20 s, with a field of view of 15 mm × 13 mm. The use of polyurethane was based on the American Society for Testing and Materials’ (ASTM F-1839-08 [28]) approval of the use of polyurethane for testing instruments and dental implants (“Standard Specification for Rigid Polyurethane Foam for Use as a Standard Material for Test Orthopedic Devices for Instruments”) [29]. Afterwards, the bicortical orthodontic self-drilling mini-implants (Biomaterials Korea, Seoul, Republic of Korea) were randomly assigned (Epidat 4.1, Galicia, Spain) to one of the following study groups: Group A. Bicortical orthodontic self-drilling mini-implants placement by a computer-aided static navigation technique (NemoScan^®^, Nemotec, Madrid, Spain) (NAV) (*n* = 40), Group B. Bicortical orthodontic self-drilling mini-implants placement by an augmented reality device (Hololens2, Redmond, WA, USA) (AR) (*n* = 40) and Group C. Bicortical orthodontic self-drilling mini-implants placement by conventional freehand technique (FHT) (*n* = 40).

### 2.3. Computer-Aided Static Navigation Technique

**Figure 1:**
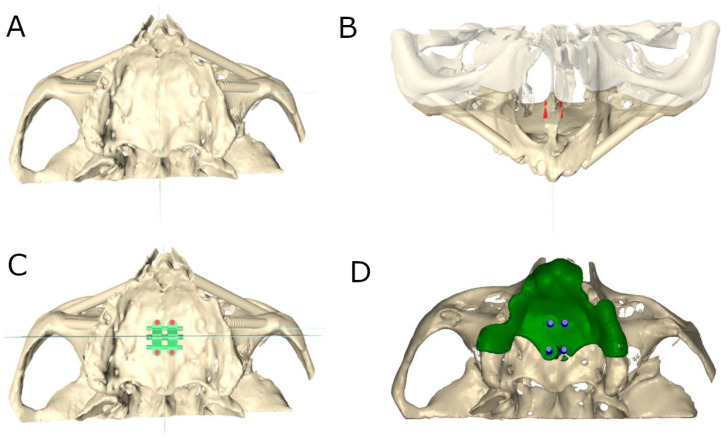
(**A**) Occlusal view of preoperative digital file, (**B**) Simulated bone marking (green) front view of bicortical orthodontic self-drilling mini-implants virtually planned, (**C**) occlusal view of SME appliance virtually planned and (**D**) surgical template virtually planned.

Subsequently, bicortical orthodontic self-drilling mini-implants (Biomaterials Korea, Seoul, Republic of Korea) and three fixation mini-implants (one anterior and two posterior) to the buccal cortical plate were virtually planned using 3D implant-planning software (Ortosan, Madrid, Spain) with the aforementioned measurements (Figure 1A–C). Afterwards, the virtual templates were also designed (Figure 1D) and manufactured using stereolithography (ProJet 6000, 3D Systems, Rock Hill, SC, USA). The stability of all surgical templates was checked, and no adjustment was necessary.

**Figure 2:**
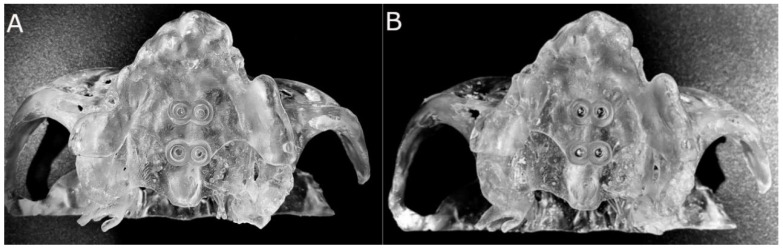
(**A**) Occlusal view of anatomically based standardized polyurethane models of a completely edentulous upper maxilla with stereolithographic surgical template and (**B**) bicortical orthodontic self-drilling mini-implants on palate.

Finally, bicortical orthodontic self-drilling mini-implants (Biomaterials Korea, Seoul, Republic of Korea) were placed using the mini-screw driver (Edison Medical, San Antonio, TX, USA) according to the technique used in MARPE and comprises the insertion of four bicortical orthodontic self-drilling mini-implants adjacent to the midpalatal suture, being two mesial and two distal to the expanding screw. Bicortical orthodontic self-drilling mini-implants were chosen taking into consideration the anatomical characteristics of the palate area. The mean thickness of bone in the regions mesial and distal to the expanding screw varies, respectively, from 3.77 to 3.88 mm and from 2.33 to 2.44 mm (Figure 2A,B).

### 2.4. Augmented Reality Technique

**Figure 3:**
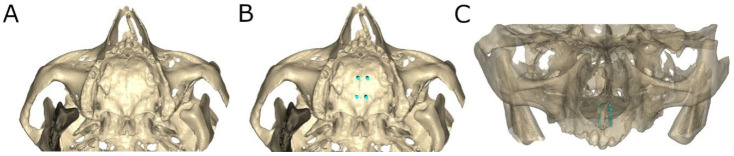
(**A**) Rendered occlusal view of preoperative STL digital file, (**B**) rendered occlusal view of bicortical orthodontic self-drilling mini-implants (blue) virtually planned and (**C**) rendered frontal view of orthodontic self-drilling mini-implants virtually planned.

The bicortical orthodontic self-drilling mini-implants implants (Biomaterials Korea, Seoul, Republic of Korea) randomly assigned to the AR study group were virtually planned using the 3D implant-planning software (Ortosan, Madrid, Spain) with the measures previously described (Figure 3A–C).

**Figure 4:**
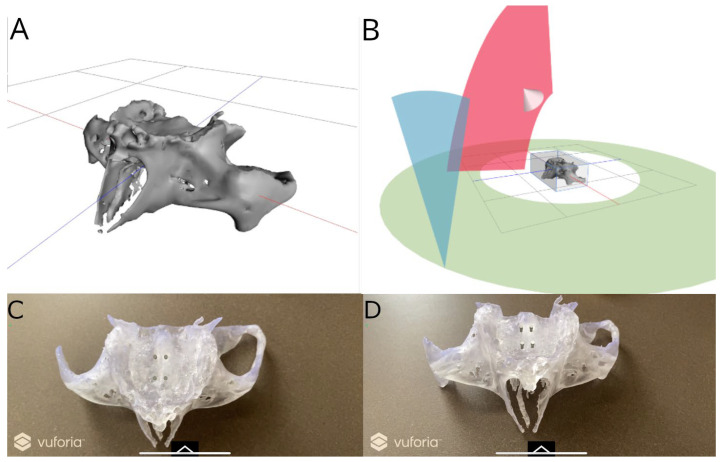
(**A**,**B**) Planning process in multi-platform augmented reality and mixed reality application development platform and (**C**) occlusal and (**D**) frontal view of anatomically based standardized polyurethane models with virtually planned bicortical orthodontic self-drilling mini-implants (grey).

Afterwards, the STL digital file of the bicortical orthodontic micro-screws designed in the 3D implant-planning software (Ortosan, Madrid, Spain) were exported to a multi-platform augmented reality and mixed reality application development platform (Vuforia, Vuforia Engine 10.13 (2023) Unity Technologies, San Francisco, CA, USA) to allow the identification, tracking and alignment of the STL digital file of the bicortical orthodontic micro-screws on the anatomically based standardized polyurethane models of a completely edentulous upper maxilla, by the recognition of key points (alveolar ridge, zygomatic arch, and anterior nasal spine) of the anatomically based models (Figure 4A,B). Afterwards, the multi-platform augmented reality and mixed reality application development platform (Vuforia, Unity Technologies) was installed in an augmented reality appliance (Hololens2, Redmond, WA, USA). Finally, the STL digital file of the bicortical orthodontic micro-screws was uploaded in the application to visualize the STL digital file on the orography of the anatomically based standardized polyurethane models of a completely edentulous upper maxilla (Figure 4C,D).

Finally, bicortical orthodontic self-drilling mini-implants (Biomaterials Korea, Seoul, Republic of Korea) were placed with the mini-screw driver (Edison Medical, San Antonio, TX, USA). Using the augmented reality digital system the clinician can visualize the surgical field in real-time. Then, the implant planned position and the bone anatomy around the implant site are checked in real time during the whole surgical procedure while mini-implants are placed in the indicated position by augmented reality system.

### 2.5. Conventional Freehand Technique

The bicortical orthodontic self-drilling mini-implants (Biomaterials Korea, Seoul, Republic of Korea) randomly assigned to the NAV, AR and FHT study group were placed in the anatomically based standardized polyurethane models of a completely edentulous upper maxilla by a unique operator who was allowed access to the planning.

Researchers randomized the order of placement of the bicortical orthodontic self-drilling mini-implants (Biomaterials Korea, Seoul, Republic of Korea), according to the placement site in palate (Epidat 4.1, Galicia, Spain), and assigned them to one of the following groups: (1) orthodontic self-drilling mini-implants (Biomaterials Korea, Seoul, Republic of Korea) placed on the right posterior site in palate (*n* = 10); (2) orthodontic self-drilling mini-implants (Biomaterials Korea, Seoul, Republic of Korea) placed on right anterior site in palate (*n* = 10); (3) orthodontic self-drilling mini-implants (Biomaterials Korea, Seoul, Republic of Korea) placed on the left anterior site in palate (*n* = 10); (4) orthodontic self-drilling mini-implants (Biomaterials Korea, Seoul, Republic of Korea) placed on the left posterior site in palate (*n* = 10).

Finally, bicortical orthodontic self-drilling mini-implants (Biomaterials Korea, Seoul, Republic of Korea) were placed manually using the mini-screw driver (Edison Medical, San Antonio, TX, USA). The unique operator had access to the CBCT scan. All orthodontic self-drilling mini-implants (Biomaterials Korea, Seoul, Republic of Korea) were placed by a unique operator with prior surgical experience.

### 2.6. Measurement Procedure

**Figure 5:**
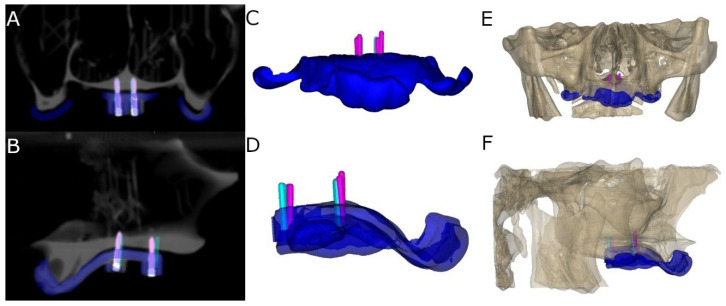
Simulated bone marking (blue): (**A**) Sagittal and (**B**) coronal view of virtually planned (blue cylinders) and performed orthodontic self-drilling mini-implants (green cylinders) and surgical template (green line) on CBCT scan, (**C**) top and (**D**) front view of virtually planned (blue cylinders) and performed orthodontic self-drilling mini-implants (green cylinders) and surgical template and (**E**) front view and (**F**) sagittal view of virtually planned (blue cylinders) and performed orthodontic self-drilling mini-implants (green cylinders) and surgical template in completely edentulous upper maxilla.

Following placement of the bicortical orthodontic self-drilling mini-implants (Biomaterials Korea, Seoul, Republic of Korea), the researchers conducted postoperative CBCT scans (WhiteFox, Satelec, Merignac, France) using the aforementioned exposure parameters (Figure 5A,B). The planning and postoperative CBCT scans (WhiteFox, Satelec, Merignac, France) of the different groups were subsequently imported into 3D implant-planning software (NemoScan, Nemotec, Madrid, Spain). The scans were then overlaid to assess the deviation, measured at the coronal entry point (mm), apical end point (mm) and angular deviation (°), with the latter measured at the center of the cylinder; according to the measurement procedure established by Van Assche et al. [30]. Any deviations that were noted in any of the bicortical orthodontic self-drilling mini-implants (Biomaterials Korea, Seoul, Republic of Korea) were subsequently analyzed and compared between the axial, sagittal and coronal views (Figure 5C–F) by an independent operator. All these measurement procedures were performed according to the methods conducted in a previous study [31].

Finally, the time (sec) necessary to place each bicortical orthodontic self-drilling mini-implants was also registered.

### 2.7. Statistical Tests

Statistical analysis was carried out using SAS 9.4 (SAS Institute Inc., Cary, NC, USA). The mean and standard deviation (SD) were used for descriptive analysis of quantitative data. Mixed linear models were fitted for each of the response variables. The individual has been included as a random factor and the variables group, implant and their interaction as fixed factors. In case of detecting statistically significant differences between times, Student *t*-test statistical analysis was performed. The *p*-values were adjusted using the Tukey method to correct the type I error. *p* < 0.05 was determined as the level for statistical significance.

## 3. Results

Table 1 shows the mean, median and standard deviation values for the coronal entry point (mm), apical end point (mm) and angular deviations (°) of each bicortical orthodontic self-drilling mini-implant.

**Figure 6:**
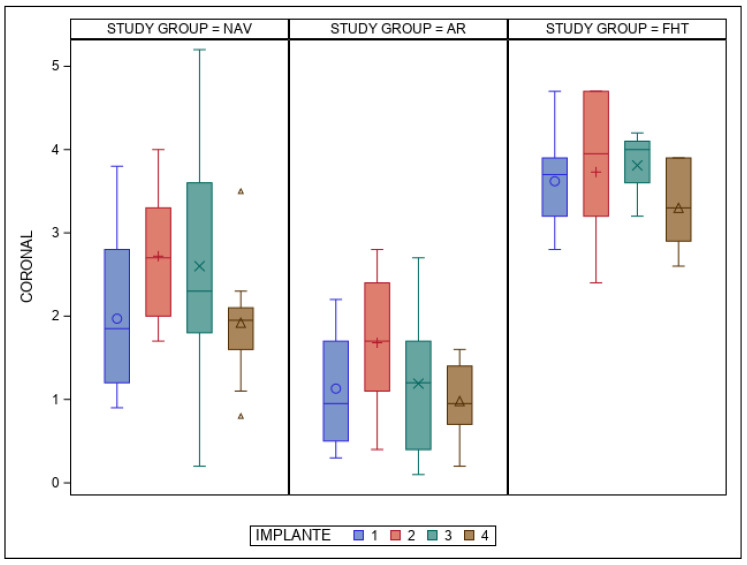
Box plot of mean and SD values of each orthodontic self-drilling mini-implants at coronal entry-point. The horizontal line in each box represents the respective median value of the study groups. +, o, x, Δ; Mean value of box plots. IOI 1: orthodontic self-drilling mini-implants placed on right posterior site in palate; IOI 2: orthodontic self-drilling mini-implants placed on right anterior site in palate; IOI 3: orthodontic self-drilling mini-implants placed on left anterior site in palate; IOI 4: orthodontic self-drilling mini-implants placed on left posterior site in palate.

The paired *t*-test found statistically significant differences (*p* < 0.001) between the NAV, AR and FHT orthodontic self-drilling mini-implant placement techniques at the coronal entry-point deviations (Figure 6). Additionally, statistically significant differences (*p* = 0.004) were also shown between the orthodontic self-drilling mini-implant positions.

**Figure 7:**
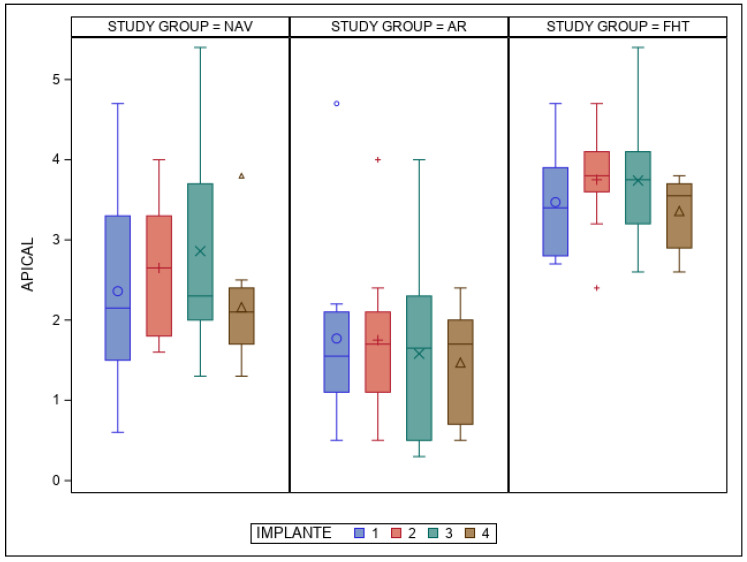
Box plot of mean and SD values of each orthodontic self-drilling mini-implants at the apical end-point. The horizontal line in each box represents the respective median value of the study groups. +, o, x, Δ; Mean value of the box plots. IOI 1: orthodontic self-drilling mini-implants placed on right posterior site in palate; IOI 2: orthodontic self-drilling mini-implants placed on right anterior site in palate; IOI 3: orthodontic self-drilling mini-implants placed on left anterior site in palate; IOI 4: orthodontic self-drilling mini-implants placed on left posterior site in palate.

The paired *t*-test found statistically significant differences (*p* < 0.001) between the NAV, AR and FHT orthodontic self-drilling mini-implant placement techniques at the apical end-point deviations (Figure 7). However, statistically significant differences (*p* = 0.270) were not found between the orthodontic self-drilling mini-implant positions (Figure 7).

**Figure 8:**
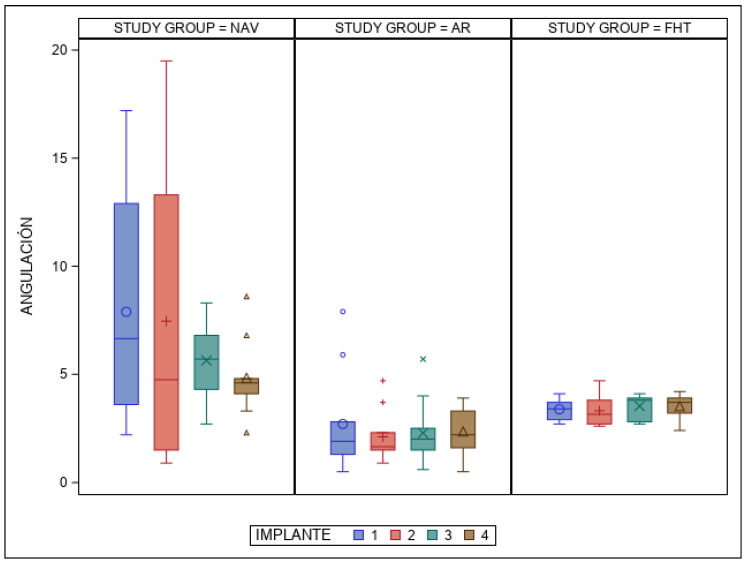
Box plot of mean and SD values of each orthodontic self-drilling mini-implants at the angular deviations. The horizontal line in each box represents the respective median value of the study groups. +, o, x, Δ; Mean value of the box plots. IOI 1: orthodontic self-drilling mini-implants placed on right posterior site in palate; IOI 2: orthodontic self-drilling mini-implants placed on right anterior site in palate; IOI 3: orthodontic self-drilling mini-implants placed on left anterior site in palate; IOI 4: orthodontic self-drilling mini-implants placed on the posterior site in palate.

The paired *t*-test found statistically significant differences (*p* < 0.001) between the NAV, AR and FHT orthodontic self-drilling mini-implant placement techniques at the angular deviations (Figure 8). In addition, statistically significant differences (*p* = 0.004) were also found between the orthodontic self-drilling mini-implant positions (Figure 8).

Table 2 shows the mean, median and standard deviation values for the time (sec) required to place each bicortical orthodontic self-drilling mini-implant with the different placement techniques.

**Figure 9:**
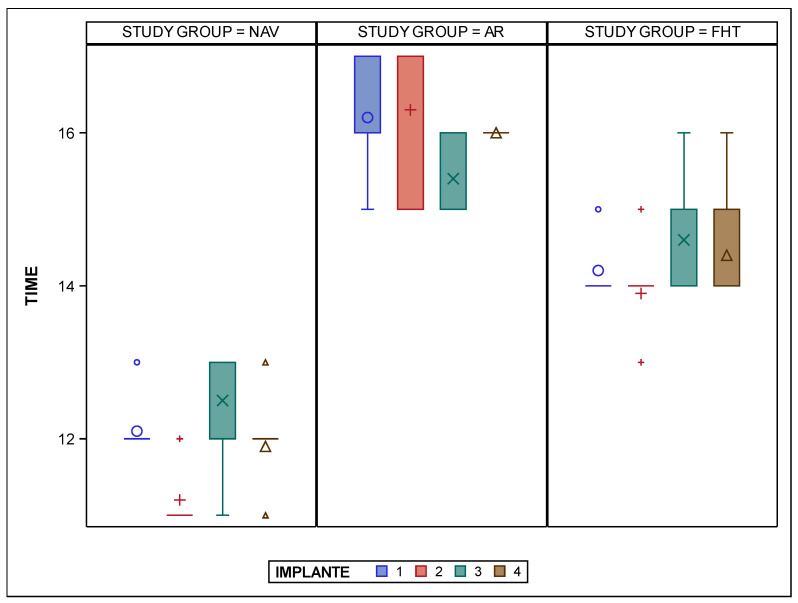
Box plot of mean and SD values of the time (sec) required to place each bicortical orthodontic self-drilling mini-implant with the different placement techniques. The horizontal line in each box represents the respective median value of the study groups. +, o, x, Δ; Mean value of box plots. IOI 1: orthodontic self-drilling mini-implants placed on right posterior site in palate; IOI 2: orthodontic self-drilling mini-implants placed on right anterior site in palate; IOI 3: orthodontic self-drilling mini-implants placed on left anterior site in palate; IOI 4: orthodontic self-drilling mini-implants placed on left posterior site in palate.

The paired *t*-test found statistically significant differences (*p* < 0.001) between the time (sec) required to place each bicortical orthodontic self-drilling mini-implant with the different placement techniques (Figure 9). Additionally, statistically significant differences (*p* = 0.004) were also shown between the orthodontic self-drilling mini-implant positions.

## 4. Discussion

The results of the present study reject the null hypothesis (H_0_) that there are no differences in the accuracy of the computer-aided static navigation technique, augmented reality and freehand placement technique for the bicortical orthodontic self-drilling mini-implants for MSE appliances. Additionally, statistically significant differences were also shown at the coronal entry-point and angular deviations between planned and performed placement site of the orthodontic self-drilling mini-implants positions.

MARPE is a promising method for correction of transversal maxillary deficiencies in adult patients. A surgical guide can provide an accurate transfer from digital planning to the surgical area without compromising the surgeon’s vision or access [32].

Planning for treatment with MARPE consists of two steps. First, suture evaluation in order to assess the degree of synostosis in the palatal suture, and therefore the possibility of expansion, and secondly, the three-dimensional positioning of both expander device and mini-implants in the palatal area [33]. A large number of studies have demonstrated that the palate is a suitable site for skeletal anchorage [14,34,35] and mini-implants placed in the paramedian palate in order to support orthodontic devices have proven to have excellent success rates as well [36]. Although the palate is a safe mini-screw site due to the absence of dental roots, it does not present uniform thickness, which makes bone assessment analysis an important phase of MARPE [37,38]. Therefore, estimation of bone availability before mini-screw insertion can assist in primary stability and lower mini-implants failure rate [39].

A 3D surgical guide can help the clinician avoid anatomical sites during mini-implant insertion as well as relieve discomfort of the patient and, as a result, the procedure becomes simpler and faster [40]. According to a study by Bae et al., in which the authors placed mini-screws in cadaver maxillae with surgical guide assistance, mini-screws were found to be placed more accurately when surgical guides were used rather than with direct method [41]. More specifically, in the surgical guide group, the angular deviation was a median of 3.14° (range, 1.02–10.9°), and the mesiodistal deviations in the coronal and apical areas were medians of 0.29 mm (range, 0.03–0.73 mm) and 0.21 mm (range, 0.03–0.97 mm), respectively. In the present study, the mean angular deviation of the orthodontic self-drilling mini-implants placed by a computer-aided static navigation technique (NAV) on the right posterior site in palate (IOI 1) was 7.89°, the mean angular deviation of the orthodontic self-drilling mini-implants placed by a computer-aided static navigation technique (NAV) on the right anterior site in palate (IOI 2) was 7.46°, the mean angular deviation of the orthodontic self-drilling mini-implants placed by a computer-aided static navigation technique (NAV) on the left anterior site in palate (IOI 3) was 5.64° and the mean angular deviation of the orthodontic self-drilling mini-implants placed by a computer-aided static navigation technique (NAV) on the left posterior site in palate (IOI 4) was 4.83°. However, no statistically significant differences were shown between the IOI 1, IOI 2, IOI 3 and IOI 4 placed by a computer-aided static navigation technique (NAV) (*p* = 0.336). In a study mentioned above, Liu et al. found that the angular deviation of mini-implants was 1.2° ± 0.43°, and the mesiodistal deviation was 0.42 ± 0.13 mm at the apical area when placed with surgical guides [11]. Morea et al. reported that the angular deviation was 1.76°, and the 3D linear distance deviations were 0.86 mm (coronal) and 0.87 mm (apex) using surgical guides [12]. However, the surgical guides that they used differ significantly from the one presented in this study as they covered more than two quadrants or all teeth and mini-implants were placed in the interradicular space and not in palate. Recently, Cantarella et al. proposed guidelines for MARPE by using digital model of dental arches and CBCT. After a virtual model of maxillary skeletal expansion (MSE) appliance with the four micro-implants was created a positioning guide is virtually designed, 3D printed and utilized to model and weld the MSE supporting arms to the molar bands. Then, the appliance is placed in the oral cavity and mini-implants are inserted [27]. In another study Lo Giudice et al. used the method of Cantarella adding to it the patient’s CBCT DICOM file that allows discriminating between cortical and cancellous bone [42]. A systematic review of nine different computer-assisted (static) guided implant systems found that the clinical performance of those systems was highly accurate with an implant survival rate of 97.3% after 12 months of observation in different indications. The meta-analysis revealed a mean error of 1.12 mm at the entry point and 1.39 mm at the apex of the in vitro and in vivo studies. In addition, it was found that the tooth- and mucosa-supported guides may have a better accuracy in comparison to the bone-supported guides [19]. Moreover, Kivovics et al. aimed to compare the accuracy of implant placement in model surgeries carried out by implementation of augmented reality based dynamic navigation, free-hand method and computer-assisted implant surgery and concluded that implant positioning accuracy of AR-based dynamic navigation was comparable to that of static CAIS and superior to that obtained by the free-hand approach [43]. In addition, Pellegrino et al. evaluated the feasibility of using a virtual display for dynamic navigation via AR and evaluate if the use of this technology could affect the accuracy of dynamic navigation [44]. Unfortunately implant surgery is prone to complications. The results of both static and dynamic digital techniques are promising but further investigation is needed to establish more data. Augmented intelligence is an important tool for operators and, due to the rapid digital advance, it can be used to many fields through intraoperative navigation. Results have shown that augmented reality provides surgeons with better chances of achieving good results; however, the cost is still an issue to be solved as it is an expensive tool for daily practice in dental clinics [22].

In addition, Riad Deglow et al. (2023) compared the accuracy and root contact prevalence between conventional freehand technique and two navigation techniques based on augmented reality technology for the orthodontic self-drilling mini-implants placement, and reported that the navigation techniques based on augmented reality technology showed fewer intraoperative complications, comparing to the conventional free-hand technique [45]. However, recent literature highlights the importance of the geometric design [46,47,48,49] and surface treatment of orthodontic micro-screws in their stability [50,51]. The authors agree in highlighting the importance of these factors in the outcome of orthodontic treatment; however, it should not be forgotten that the placement technique for orthodontic mini-implants influences their precision and the occurrence of intraoperative complications. Therefore, clinicians are encouraged to pay attention to all parameters to ensure treatment success while minimizing risks.

This study has certain limitations inherent of in vitro research on the physical properties and setting conditions of research materials. Specifically, it is challenging to replicate all the patient-related factors that can affect the results, making it difficult to directly apply the findings to real world clinical scenarios. However, basic research is useful in order to control all parameters and isolate the variable of study. In future investigations, it would be valuable to reproduce the methodological design clinically to determine whether the setting conditions influences the mechanical behavior of the orthodontic mini-implants. Specifically, the authors encourage future researchers to conduct clinical studies with different operators and different bone densities to analyze the influence of these variables on the accuracy of the orthodontic mini-implants placement techniques for MSE appliances.

Additionally, the strength of the present study is based on the development of a novel and innovative application based on extended reality. The accuracy of this technology was compared for the orthodontic mini-implants placement for MSE appliances with a computer-aided static navigation technique through a surgical template (as gold standard). Additionally, the authors used the freehand placement technique as a control group.

The present study was performed under controlled in vitro conditions and orthodontic self-drilling mini-implants were placed by a certified surgeon with ten years of experience. However, further studies should be performed, preferably in vivo, so that the results can be reinforced.

## 5. Conclusions

The results from the augmented reality placement technique demonstrate superior accuracy, providing the most precise positioning of MSE appliances on the palate. Empirical data indicate that this method outperforms other techniques in terms of precision. The computer-aided static navigation technique follows closely, showing notable improvements in accuracy compared to traditional approaches. In contrast, the freehand technique, while still commonly used, yields the least precise outcomes in the placement of MSE appliances on the palate, as evidenced by higher measurement errors and deviations from the intended positioning. These findings underscore the effectiveness of augmented reality in enhancing surgical and orthodontic procedures, particularly in the context of MSE appliance placement.

## Figures and Tables

**Table 1:** Descriptive values of deviations at coronal entry point (mm), apical end point (mm) and angular (°) deviations of each orthodontic self-drilling mini-implant.

Study Group	Measure	IOI	*n*	Mean	SD	Median	Minimum	Maximum
NAV	Coronal	1	10	1.97	0.95	1.85	0.90	3.80
		2	10	2.72	0.77	2.70	1.70	4.00
		3	10	2.60	1.40	2.30	0.20	5.20
		4	10	1.92	0.72	1.95	0.80	3.50
	Apical	1	10	2.36	1.31	2.15	0.60	4.70
		2	10	2.65	0.85	2.65	1.60	4.00
		3	10	2.86	1.26	2.30	1.30	5.40
		4	10	2.16	0.70	2.10	1.30	3.80
	Angular	1	10	7.89	5.19	6.65	2.20	17.20
		2	10	7.46	6.71	4.75	0.90	19.50
		3	10	5.64	1.64	5.70	2.70	8.30
		4	10	4.83	1.75	4.60	2.30	8.60
AR	Coronal	1	10	1.13	0.73	0.95	0.30	2.20
		2	10	1.68	0.76	1.70	0.40	2.80
		3	10	1.19	0.87	1.20	0.10	2.70
		4	10	0.98	0.45	0.95	0.20	1.60
	Apical	1	10	1.77	1.15	1.55	0.50	4.70
		2	10	1.75	1.00	1.70	0.50	4.00
		3	10	1.58	1.17	1.65	0.30	4.00
		4	10	1.47	0.71	1.70	0.50	2.40
	Angular	1	10	2.70	2.37	1.90	0.50	7.90
		2	10	2.11	1.18	1.65	0.90	4.70
		3	10	2.28	1.57	2.00	0.60	5.70
		4	10	2.36	1.07	2.20	0.50	3.90
FHT	Coronal	1	10	3.62	0.57	3.70	2.80	4.70
		2	10	3.73	0.89	3.95	2.40	4.70
		3	10	3.81	0.38	4.00	3.20	4.20
		4	10	3.30	0.56	3.30	2.60	3.90
	Apical	1	10	3.47	0.68	3.40	2.70	4.70
		2	10	3.75	0.61	3.80	2.40	4.70
		3	10	3.74	0.81	3.75	2.60	5.40
		4	10	3.36	0.43	3.55	2.60	3.80
	Angular	1	10	3.38	0.46	3.40	2.70	4.10
		2	10	3.31	0.70	3.15	2.60	4.70
		3	10	3.53	0.57	3.80	2.70	4.10
		4	10	3.53	0.59	3.70	2.40	4.20

Statistically significant differences (*p* < 0.05) found between groups 1, 2, 3 and 4 and between NAV, AR and FHT study groups. Group IOI 1: orthodontic self-drilling mini-implants placed on right posterior orifice of surgical template. Group IOI 2: orthodontic self-drilling mini-implants placed on right anterior orifice of surgical template. Group IOI 3: orthodontic self-drilling mini-implants placed on left anterior orifice of surgical template. Group IOI 4: orthodontic self-drilling mini-implants placed on left posterior orifice of surgical template.

**Table 2:** Descriptive values of time (sec) required to place each bicortical orthodontic self-drilling mini-implant with different placement techniques.

Study Group	IOI	*n*	Mean	SD	Median	Minimum	Maximum
NAV	1	10	12.10	0.32	12.00	12.00	13.00
	2	10	11.20	0.42	11.00	11.00	12.00
	3	10	12.50	0.85	13.00	11.00	13.00
	4	10	11.90	0.57	12.00	11.00	13.00
AR	1	10	16.20	0.63	16.00	15.00	17.00
	2	10	16.30	0.95	17.00	15.00	17.00
	3	10	15.40	0.52	15.00	15.00	16.00
	4	10	16.00	0.00	16.00	16.00	16.00
FHT	1	10	14.20	0.42	14.00	14.00	15.00
	2	10	13.90	0.57	14.00	13.00	15.00
	3	10	14.60	0.84	14.00	14.00	16.00
	4	10	14.40	0.70	14.00	14.00	16.00

## Data Availability

The datasets used and/or analyzed during the current study are available from the corresponding author on reasonable request.
